# Measurement of Human Papillomavirus-Specific Antibodies Using a Pseudovirion-Based ELISA Method

**DOI:** 10.3389/fimmu.2020.585768

**Published:** 2020-10-28

**Authors:** Zheng Quan Toh, Laura He, Catherine Chen, Angela Huang, Fiona M. Russell, Suzanne M. Garland, Rita Reyburn, Tupou Ratu, Evelyn Tuivaga, Ian H. Frazer, E. Kim Mulholland, Paul V. Licciardi

**Affiliations:** ^1^New Vaccines, Murdoch Children’s Research Institute, Infection and Immunity, Parkville, VIC, Australia; ^2^Department of Paediatrics, The University of Melbourne, Parkville, VIC, Australia; ^3^Department of Obstetrics and Gynecology, The University of Melbourne, Parkville, VIC, Australia; ^4^Regional WHO HPV Reference Laboratory, Centre Women’s Infectious Diseases Research, The Royal Women’s Hospital, Parkville, VIC, Australia; ^5^Public Health Services, Ministry of Health and Medical Services, Suva, Fiji; ^6^Faculty of Medicine, Diamantina Institute, The University of Queensland, Brisbane, QLD, Australia; ^7^Department of Infectious Disease Epidemiology, London School of Hygiene and Tropical Medicine, London, United Kingdom

**Keywords:** human papillomavirus (HPV), ELISA—enzyme-linked immunosorbent assay, antibody, pseudovirion, subtypes

## Abstract

Human papillomavirus (HPV) vaccines are safe and effective in preventing HPV infection and cervical precancers. Neutralizing antibodies are thought to be the primary mechanism of protection for HPV vaccines, although the exact level required for protection has not been identified. Three common serological assays used in clinical trials to measure HPV antibodies are HPV pseudovirion-based neutralization assay (PBNA), competitive or total Luminex immunoassays (cLIA or LIA) and VLP-based enzyme linked immunosorbent assays (ELISA). While PBNA is the gold-standard for measuring neutralizing antibodies (NAb), it is labor intensive. Luminex immunoassay and VLP-ELISA are rapid and high throughput, but their reagents and equipment can be difficult to source. Nevertheless, data generated from these assays generally correlate well with PBNA. Here, we described a simplified high-throughput PsV-based ELISA for HPV antibody measurement, to circumvent some of the limitations of existing assays. Using this assay, we were able to differentiate HPV-specific IgG and IgM, and found a strong correlation between HPV-specific IgG and NAb levels, as previously determined by PBNA. This assay platform is simpler and less time-consuming than PBNA. In addition, the materials can be readily produced and obtained commercially. This assay can be used as an alternative method to measure HPV antibodies.

## Introduction

Three licensed virus-like particle (VLP)-based human papillomavirus (HPV) vaccines (Gardasil, Cervarix, Gardasil9) are highly immunogenic, safe and effective in preventing vaccine-type HPV infections and cervical cancer precursors (1). These prophylactic vaccines are given as three-dose schedules to individuals aged 15 to 26 years. A two-dose schedule separated by 6 months is recommended to those aged 9 to 14 years based on noninferiority studies. However, for immunosuppressed individuals, the recommendation is to receive three doses of the HPV vaccine (2). Recent observational data suggests that one dose of HPV vaccine is immunogenic and can produce antibodies that persist for at least 7 years and may be protective against vaccine-type HPV infection and HPV-related diseases (3–5). The results of ongoing randomized clinical trials of single dose HPV schedules are greatly anticipated (3).

Neutralizing antibodies (NAb) are thought to be the primary mechanism of protection for HPV vaccines, although the exact level required for protection has not been defined as there have been no breakthrough cases identified to date. Three serological assays are commonly used in clinical trials to measure HPV antibodies. They are HPV pseudovirion-based neutralization assay (PBNA), competitive or total Luminex immunoassays (cLIA or LIA) and VLP-based enzyme linked immunosorbent assays (ELISA) (6). The PBNA are considered the “gold standard” for assessing protective total neutralizing antibodies induced by the vaccines. It is a chemiluminescent-based assay utilizing HPV pseudovirions (PsV) (self-assembled HPV L1 or L1-L2 proteins and a reporter gene produced through molecular biology technology) and human embryonic kidney cell line (HEK293TT/293FT cells), as described previously (7). cLIA or LIA is a multiplex-bead based assay that measures neutralizing monoclonal antibodies that compete with an individual’s serum antibodies for binding to a specific immunodominant epitope or whole HPV VLPs epitopes, respectively (8, 9). Although cLIA is highly specific, it only measures NAbs competing for one epitope, which means that only a subset of total NAb are detected. In contrast, VLP-based ELISAs measures all neutralizing and non-neutralizing antibodies, which may overestimate the antibody response. Nevertheless, all three assays generally correlate well, particularly in specimens with high antibody levels (6).

**Figure 1:**
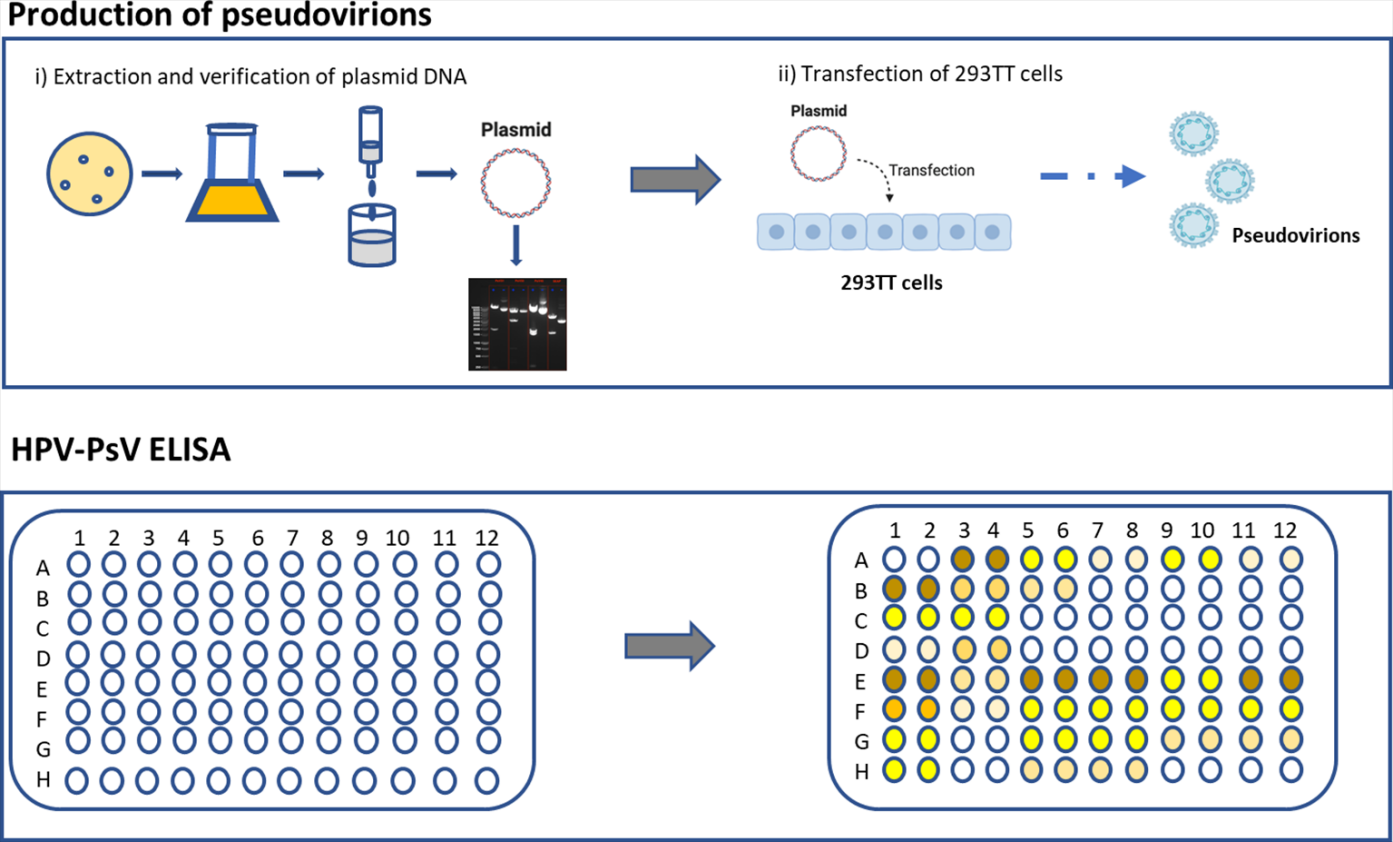
Protocol overview.

While PBNA is the gold-standard, the assay is difficult to set up and laborious. Furthermore, PBNA does not discriminate between different antibody isotypes and subclasses. cLIA/LIA and VLP-ELISA are rapid and high throughput, but the reagents and equipment are difficult to source. To establish a simpler and easier method of measuring HPV-specific antibodies, as well as the potential to measure different antibody characteristics (i.e., different isotypes and subclasses), we established an HPV PsV-based ELISA to measure HPV-specific IgG and IgM antibody responses. In this report, we provide detailed protocols for the production of HPV pseudovirions (Part I), as well as for setting up the ELISA that we have developed (Part II) (**Figure 1**). We believe that this protocol will be useful for laboratories that are examining or planning to set up HPV serology capacity. The described protocol setup works well for us, but it can easily be modified and adapted to each laboratory needs and improved by the research community in the future.

## Materials and Equipment Methods

The reagents/materials and the equipments to conduct this assay are listed in **Tables 1** and **2**, respectively.

**Table 1:** Reagents/Materials.

Purpose	Name	Manufacturer	Location
**Plasmid extraction and validation**	HPV plasmids	Addgene	Cambridge, USA
	Qiagen Maxiprep kit	Qiagen	Hilden, Germany
	Luria-Bertani (LB) media	Oxoid	VIC, Australia
	Agarose	Oxoid	VIC, Australia
	*Antibiotics	Sigma-Aldrich	MO, USA
	*Restriction enzymes		
**Production of HPV pseudovirions**	HEK293TT or FT cells	Thermofisher Scientific	VIC, Australia
	Dulbecco’s Modified Eagle’s Medium (DMEM)	Gibco Life Technologies	NY, USA
	Fetal Bovine Serum (FBS)	Hyclone GE Healthcare Life Sciences	UT, USA
	Minimum Non-Essential Amino Acid (Minimum NEAA)	Gibco Life Technologies	NY, USA
	Glutamax	Gibco Life Technologies	NY, USA
	Penicillin-Streptomycin-Glutamine (PSG)	Gibco Life Technologies	NY, USA
	Phosphate-Buffered Saline (PBS)	Gibco Life Technologies	NY, USA
	Trypsin	Gibco Life Technologies	NY, USA
	Lipofectamine	Invitrogen	CA, USA
	Opti-MEM^®^	Gibco Life Technologies	NY, USA
	RNAse cocktail	Ambion	CA, USA
	Brij	Sigma-Aldrich	MO, USA
	50-ml centrifuge tube	Corning Incorporated	NY, USA
	T75 Flask	Corning Incorporated	NY, USA
**Protein quantification**	BCA protein assay (Cat no.: 23227)	Thermofisher Scientific	VIC, Australia
**ELISA**	Maxisorp 96-well plates	Thermofisher Scientific	VIC, Australia
	PBS-0.05%Tween	Sigma-Aldrich	MO, USA
	Mouse anti-human IgG or IgM monoclonal antibodies	Southern Biotech	AL, USA
	3,3′,5,5′-tetramethylbenzidine	KPL	MA, USA
	1M Phosphoric Acid	Merck	VIC, Australia

*Antibiotics and restriction enzymes are specific for each HPV type.

**Table 2:** Equipment.

Name	Manufacturer	Location
**Nanodrop 2000 spectrophotometer**	Thermo Scientific	VIC, Australia
**ELISA Plate Reader**	BioTek Instruments	VT, USA
**Centrifuge**	LAF Technologies	VIC, Australia
**Ultra-centrifuge**	Beckman Coulter	IN, USA
**Biosafety Cabinet**	Sigma-Aldrich	Munich, Germany

### Maintenance of HEK293TT Cells

The HEK293TT cells (human embryonic kidney cell line) were kindly provided by Prof Ian Frazer’s laboratory (The University of Queensland Diamantina Institute, Australia) under a Materials Transfer Agreement. A cryovial of HEK293TT cells was thawed by adding DMEM media consisting of 20% heat-inactivated FBS, 1% minimum NEAA, 1% Glutamax and 1% PSG slowly in dropwise motion and transferred into a 50-ml conical centrifuge tube. Approximately 20 ml of media was slowly added with constant flicking of tube to ensure proper mixing and then transferred into a 75cm^2^ tissue culture flask (T75). The cells were incubated at 37°C, 5% CO_2_ for 2 to 3 days before cell passage. Following thawing of HEK293TT cells, they were passaged at least twice before use in PsV production.

Cells were passaged twice weekly at 70% to 80% confluence. Cell passage began with removal of culture medium, followed by washing of cells twice with 5 ml of PBS. Trypsin (0.25%) was then added to detach cells from the flask surface and incubated at 37°C for 1 to 2 min. The cells were then resuspended in 9 ml of D10 media (DMEM medium consisting of 10% heat-inactivated FBS, 1% minimum NEAA, 1% Glutamax and 1% PSG) and split at a 1:6 or 1:12 ratio into a T75 flask depending on level of cell confluence.

### Production of HPV Pseudovirions (PsV)

#### HPV Plasmid Extraction and Validation

The HPV plasmid DNA was obtained from Addgene (Cambridge, USA) in *Escherichia coli* stab. The bacteria containing HPV plasmid 16 were cultured on Luria-Bertani (LB) agar plates and broth containing specific antibiotics. HPV16 plasmid DNA was extracted from the bacteria using Qiagen Maxiprep kit according to manufacturer’s instruction and quantified using Nanodrop 2000 spectrophotometer. HPV16 plasmid DNA was then validated *via* restriction enzyme digestion and agarose gel electrophoresis.

#### Transfection of HEK293TT Cells

Production of HPV PsV was done by transfecting HEK293TT cells with HPV16 plasmid DNA. Three days prior to transfection, the cells were prepared at a concentration of 2 x 10^6^ per 15 ml of media in T75 flask and were incubated for 3 days at 37°C and 5% CO_2_. At day 3, a mastermix of 85 μL of lipofectamine and 2 ml of Opti-MEM®, as well as 19ug of HPV16 plasmid DNA in 2 mL of Opti-MEM® were prepared for each flask and incubated at room temperature (RT) for 20 min. The plasmid DNA-Opti-MEM^®^ mixture and mastermix were then combined and incubated for a further 20 min. The mixture was then added to the flask that was prepared 3 days prior and incubated at 37°C, 5% CO_2_ for 5 h. Following incubation, media containing DNA-mastermix mixture was replaced with 15 ml of D10 media, and cells were incubated for 48 h at 37°C, 5% CO_2_.

After 48 h, cell supernatants were collected into a 50-ml tube and 2 ml of 0.25% trypsin was added to the flask followed by incubation for 2 min at 37°C, 5% CO_2_ to detach the transfected cells from the flask. To neutralise the action of trypsin and resuspend cells, 5 ml of collected cell supernatant was added to flask and the cells were then collected in 15 ml tube. The flask was rinsed with 3 ml PBS to collect as many cells as possible. Cells were then spun *via* centrifugation at 1200 rpm and 4°C for 5 min. Following that, the cells were resuspended in 10 ml PBS and centrifuged again at 1200 rpm and 4°C for 5 min. Finally, cells were resuspended in 1 ml of PBS containing 9.5 mM magnesium (PBS-Mg). Following resuspension, cells were transferred to a low-binding microfuge tube and PBS-Mg added at 1.5 times the total cell volume. RNAse cocktail and 10% Brij were also added at 1:1,000 and 1:25 of the total cell volume, respectively. The cell lysates containing the PsV were then incubated at 37°C, 5% CO_2_ overnight for maturation. The PsVs were then aliquoted, snap-frozen, and stored at −80°C until use.

### PsV-ELISA

The amount of PsV proteins were determined by BCA protein assay prior to use for ELISA. 96-well high protein-binding ELISA plates were coated with 25 µg/ml of HPV16 PsV in PBS and incubated overnight at 4°C. Plates were washed with PBS-0.05% Tween and then blocked for 1 h with 10% FCS/PBS at 37°C. Samples (1:100) were serially diluted in 10% FCS/PBS and added to the plate along with negative (10% FCS/PBS) and positive (sera from vaccinated individuals) controls and incubated for 2 h at 37°C. A standard made up of pooled sera from vaccinated individuals were serially diluted and were used to determine the sample concentration (a value of 10 ELISA units (EU)/ml was assigned to the top standard). Plates were washed with PBS-0.05%Tween three times and goat anti-human IgG-HRP (1:2500) or goat anti-human IgM-HRP (1:2500) was added. Plates were incubated for 2 h at 37°C. After another three washes with PBS-0.05%Tween, substrate solution was added for 9mins before stopping the reaction with 1M phosphoric acid. Plates were read on a microplate reader at 450nm with a reference wavelength of 630 nm.

### Study Samples

The clinical samples used were derived from a cohort study in Fiji, which has been described in detail previously (5). Briefly, 200 Fijian girls (15–19 years old) who were previously unimmunized, or immunized with one to three doses of 4vHPV (Gardasil^®^, Merck Inc.) six years earlier were recruited. A booster dose of 2vHPV (Cervarix^®^, GSK) was given to all girls. Blood was taken pre- and 28 days following 2vHPV. A subset of samples [3-dose group (N=16), 2-dose group (N=16), 1-dose group (N=18), 0-dose group (N=20)] from each 4vHPV dosage groups were used for this analysis. All samples were randomized and tested in a blinded manner.

### Statistical Analysis

Unpaired t-tests were used to compare the geometric mean of IgG/IgM levels (with 95% CIs) between girls who were unimmunized or were immunized with one to three doses of 4vHPV pre- and 28-days following 2vHPV. A nonparametric Spearman’s correlation was performed between NAb titers previously determined (5) and IgG or IgM` EU/ml. The agreement between PBNA and PsV-ELISA was calculated using the number of double-positive results plus the number of double-negative results divided by the total number of samples analyzed. All statistical analyses were performed using Graphpad Prism 7.0. A p-value < 0.05 was considered significant.

## Results

### Optimization of PsV-ELISA

We tested three different antigen coating concentrations (50, 25, and 5 µg/ml) and two secondary antibody concentrations (1:2500 and 1:5000). We found that a coating concentration of 25 µg/ml and 1:2500 gave us the highest readout with the least background (**Supplementary Figure 1**).

### Assay Testing Using Serum From Vaccinated and Unvaccinated Individuals

To verify our assay, we measured HPV16-specific IgG and IgM antibody responses using serum samples collected from a cohort study in Fiji; girls previously immunized with one to three doses of 4vHPV six years before (pre), and one month after a dose of 2vHPV (post).

**Figure 2:**
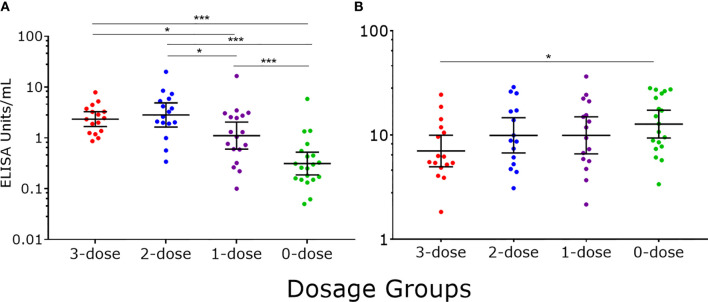
HPV16-specific antibody responses measured by PsV-ELISA six years after the last dose of 4vHPV. **(A)** HPV-specific IgG levels **(B)** HPV-specific IgM levels. Each dot represents an individual sample; error bars represent geometric mean ± 95% confidence interval. *p < 0.05, ***p < 0.001.

Six years after the last dose of 4vHPV, we detected a dose response between the different 4vHPV dosage groups, with higher IgG levels in higher dosage groups. Significantly higher IgG levels were found in girls who were previously immunized with one to three doses of 4vHPV when compared with unimmunized girls (**Figure 2A**). Statistically higher IgM levels were in unimmunized girls compared with girls previously immunized with three doses of 4vHPV (**Figure 2B**).

**Figure 3:**
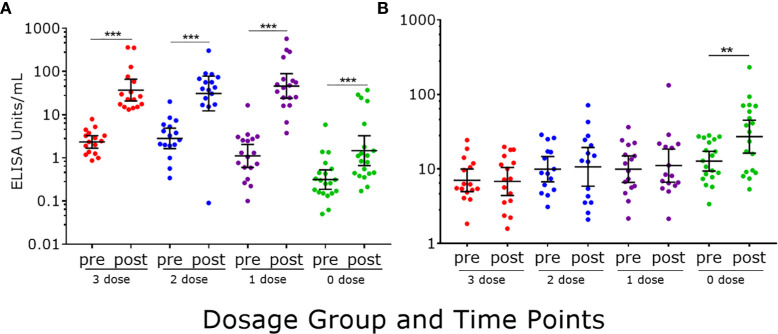
HPV16-specific antibody responses measured by PsV-ELISA pre- and post-2vHPV. **(A)** HPV-specific IgG levels **(B)** HPV-specific IgM levels. Each dot represents an individual sample; error bars represent geometric mean ± 95% confidence interval. **p < 0.01, ***p < 0.001.

Following a dose of 2vHPV, a statistically significant increase in IgG levels were detected in all dosage groups (p<0.0001 in all cases) (**Figure 3A**). Girls previously immunized with one to three doses of 4vHPV had significantly higher IgG levels than unvaccinated girls (p<0.0001 in all cases). Within dosage groups, girls previously immunized with three and two doses of 4vHPV had a 17- and 18-fold increase in IgG levels, respectively, and girls previously immunized with one dose of 4vHPV had a 79-fold increase, whilst the previously unimmunized girls had a 5-fold increase in IgG. Only girls previously unimmunized had significantly higher IgM levels (2-fold) following a dose of 2vHPV (**Figure 3B**); their level was significantly higher than girls previously immunized with three (p<0.001), two (p=0.03) and one dose (p=0.011) of 4vHPV.

We also examined antibodies to HPV18 on a subset of samples (N=26) using this assay, and similar observations as HPV16 antibody responses were found between different dosage groups (**Supplementary Figure 2**).

### Correlation and Agreement Between PsV-ELISA and PBNA

**Figure 4:**
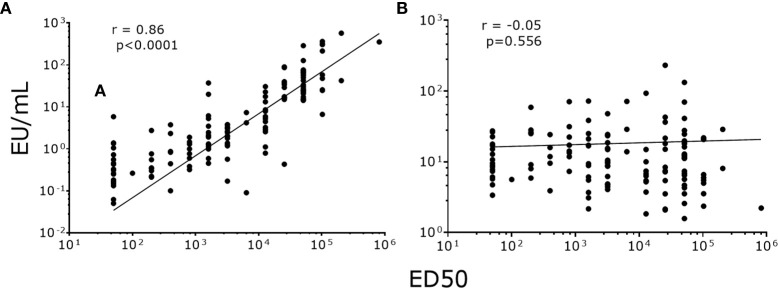
Correlation of NAb titers (ED50) produced by PBNA with antibody levels produced by PsV16-ELISA. **(A)** IgG **(B)** IgM. ED50: effective dose 50.

**Figure 5:**
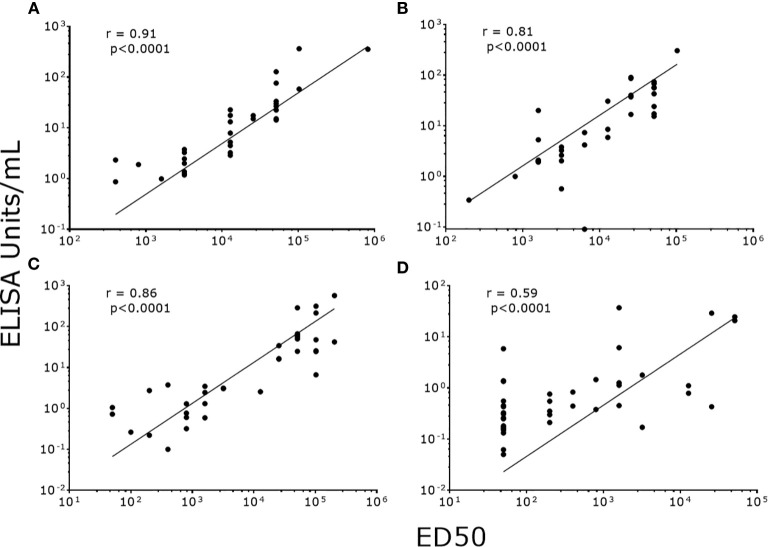
Correlation of NAb titers (ED50) produced by PBNA with IgG levels (EU/ml) produced by PsV16-ELISA stratified by dosage groups. **(A)** 3-dose group **(B)** 2-dose group **(C)** 1-dose group **(D)** 0-dose group. ED50: effective dose 50.

**Figure 6:**
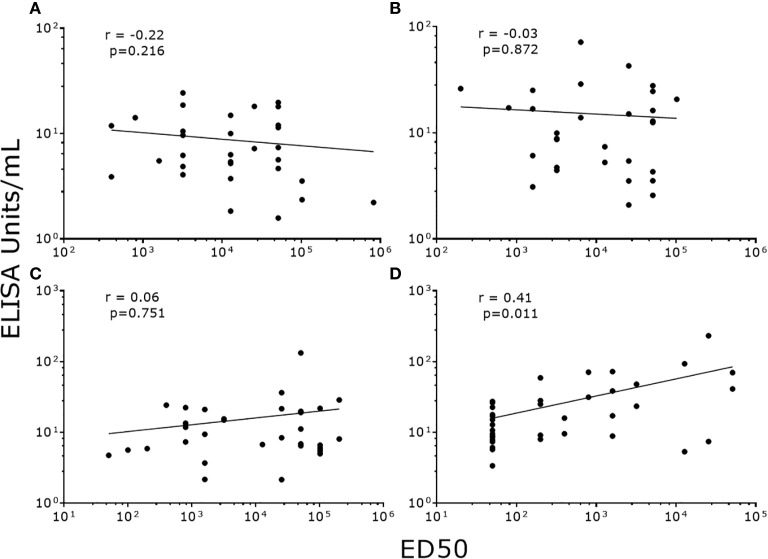
Correlation of NAb titers (ED50) produced by PBNA with IgM levels (EU/ml) produced by PsV16-ELISA stratified by dosage groups. **(A)** 3-dose group **(B)** 2-dose group **(C)** 1-dose group **(D)** 0-dose group. ED50: effective dose 50.

To validate our data, we correlated NAb titers (effective dose 50, ED50) previously determined by PBNA in our laboratory, with the values produced by PsV-ELISA. A strong statistically significant (p<0.0001) positive correlation were found between IgG levels (PsV-ELISA) and NAb titers (PBNA) (**Figure 4A**), while no correlation between IgM and NAb titers were found (**Figure 4B**). When stratified by dosage groups, strong statistically significant (p<0.0001) positive correlations were found between the IgG and NAb titers for all dosage groups (**Figure 5**), whereas only girls in the 0-dose group had a moderately significant positive correlation between the IgM and NAb titers (**Figure 6**).

The IgG seropositive cutoff for this PsV ELISA was determined using ELISA values from unvaccinated girls with negative PBNA results (N=17, excluding one outlier), and was 0.374 EU/ml. Based on this, the sensitivity and specificity for the PsV ELISA compared to PBNA is 94.0% and 56.5%, respectively (**Table 3**).

**Table 3:** Assay agreement between PsV-ELISA and PBNA.

	HPV16 PsV-ELISA		
PBNA	Positive	Negative	Total
Positive	110	10	120
Negative	7	13	20
Total	117	23	140
Sensitivity (%)	94.0		
Specificity (%)	56.5		
Agreement (%)	87.8		
Positive predictive value (%)	91.6		
Negative predictive value (%)	65.0		

## Discussion

Using a PsV-based ELISA, we were able to differentiate IgG and IgM antibodies from previously immunized and unimmunized girls. As expected, we found that the majority of NAbs were IgG in previously immunized girls, and that unimmunized girls receiving their first dose of HPV vaccine have a combination of IgG and IgM antibodies, one month following vaccination. Our results suggest that a PsV-based ELISA may be used as an alternative to the PBNA, and VLP-based ELISA for the measurement of HPV-specific antibody responses following vaccination. The main benefit of this assay is that the materials/reagents are easier to produce and/or are commercially available. The advantage of this assay over VLP-based ELISA is that the PsV does not require purification unlike VLPs. Compared to PBNA, it is simpler and quicker to perform, and thus allows for a higher throughput. Furthermore, it also has the advantage of being able to measure antibody characteristics such as isotype (IgG, IgM, IgA, and IgE) and subclasses (IgG1, IgG2, IgG3, IgG4, IgA1, and IgA2), as well as antibody avidity. The clinical relevance of these antibody characteristics is unknown, but they will provide us with a better understanding of the antibody responses of HPV vaccinated individuals, particularly in the context of a single dose HPV vaccine schedule, where the antibody levels are lower. One limitation of this assay is that it detects both neutralizing and non-neutralizing antibodies, similar to most ELISAs, which can sometimes over-estimate the antibody response. This is reflected by the high sensitivity and moderate specificity analysis observed when comparing this assay to PBNA. The moderate specificity of this assay may be due neutralizing antibodies other than IgG, as well as the presence of cross-reactive non-neutralizing antibodies generated by infection with other HPV types. It is important to note our negative control sera used to determine the seropositivity cut-off were from Fijian girls in the 0-dose group who had no detectable neutralizing antibody however we cannot rule out the possibility that other antibodies may be present (e.g., non-neutralizing). As such, our sample size was small and HPV infection status was unknown. Nevertheless, this assay is unlikely to significantly affect the analysis of the HPV IgG results, since the IgG antibody levels detected using this assay strongly correlated with the neutralizing antibody titers from PBNA, consistent with the VLP-based ELISA (10, 11).

We have observed that some clinical samples have biological interferences in the serological assays which do not dilute out with one or two dilutions. Therefore, it is important to perform several dilutions (three to four dilutions) of each sample, and in duplicate wells at least, so that the data generated are more robust. Other suggestions are standard good laboratory practices which includes performing samples from the same individuals on the same plate (e.g., pre- and post-samples) to eliminate inter-plate variations as well as appropriate controls (i.e., pooled serum controls, as well as serum with high and low antibody levels) to eliminate inter-plate and inter-assay variations. In addition, it is also important to validate new batches of PsV or produce sufficient quantity for a complete study to eliminate any potential batch variation.

The current International Standards (IS) for HPV16 and 18 that define the International Units (IU) of HPV antibodies can assist in standardizing and verifying serological assays, and can be ordered from the National Institute of Biological Standards and Controls (NIBSC, Potters Bar, UK). These standards are produced from individuals who generated antibodies to natural infection, where the antibody levels are generally low (12). An IS with a more directly comparable IU from vaccinated individuals and also to the additional 5 oncogenic types included in Gardasil^®^9 will be more representative for serological examination of the current vaccines. Such an IS is currently being developed and undergoing validation (6). This IS will be extremely useful in the global harmonization of HPV serology testing in clinical trials of HPV prophylactic vaccines.

While antibodies are thought to be the primary mechanisms of protection, memory B cells are also likely to be involved in protection, and may be a better surrogate for long-term protection. This is currently of great interest for reduced-dose HPV vaccine schedules, particularly single dose schedules. B-cell ELISPOT assays using HPV VLP antigens have been developed and evaluated for the monitoring of HPV16/18 memory B-cell responses in vaccinated women. In this case, the PsV antigens could also be adapted for memory B cell ELISPOT assay, but requires further evaluation.

In conclusion, we developed a rapid and simple PsV-based ELISA to measure serum HPV antibodies. This assay can be used as an alternative method to circumvent some of the limitations of the existing assays.

## Data Availability Statement

All datasets presented in this study are included in the article/**Supplementary Material**.

## Ethics Statement

The study samples were from a cohort study that were reviewed and approved by Fiji National Research Ethics Review Committee, Fiji National Research Committee (2014.5.FNRERC.5.SU), Royal Children’s Hospital Human Research Ethics Committee, Melbourne, Australia (34239A). Written informed consent to participate in this study was provided by the participants or participants’ legal guardian/next of kin.

## Author Contributions

PL and ZT conceptualized and designed the study. ZT, LH, CC, and AH did the experiments and performed the analyses. ZT wrote the first draft of the manuscript. LH and PL wrote sections of the manuscript. FR, RR, TR, and ET designed clinical trial study design and collected the samples which were used in this study. SG, IF, and EM provided scientific and technical advice. IF shared the HPV-pseudovirion production technology. All authors contributed to the article and approved the submitted version.

## Funding

This work was supported by the Department of Foreign Affairs and Trade of the Australian government and the Fiji Health Sector Support Program (FHSSP). FHSSP is implemented by ABT Associates on behalf of the Australian government. PL is supported by an Australian National Health and Medical Research Council (NHMRC) Career Development Fellowship. FR, SG and IF are supported by NHMRC Leadership Fellowships. This work was also supported in part by the Victorian government’s Operational Infrastructure Support Program. The funders had no role in the design of the study; in the collection, analyses, or interpretation of data; in the writing of the manuscript, or in the decision to publish the results.

## Conflict of Interest

SG has received grants through her institution from Merck and has delivered lectures and received speaking fees from MSD for work performed in her personal time and is a member of the Merck HPV Global Advisory Board. The University of Queensland as employer of IHF receives royalties from the sales of Gardasil^®^ and Cervarix^®^ vaccine.

The remaining authors declare that the research was conducted in the absence of any commercial or financial relationships that could be construed as a potential conflict of interest.

## Supplementary Material

Supplementary Figure 1 Optimization conditions for antigen coating **(A)** and secondary antibody concentration **(B)** for PsV-ELISA using PsV-16. Pooled serum sample from HPV-vaccinated individuals had a starting dilution at 1:300.

Supplementary Figure 2 HPV18-specific antibody responses measured by PsV-ELISA pre- and post-2vHPV. **(A)** HPV-specific IgG levels **(B)** HPV-specific IgM levels. Each dot represents an individual sample (3-dose group, N=5, 2-dose group, N=5, 1-dose group, N=7, and 0-dose group, N=9); error bars represent geometric mean ± 95% confidence interval. **p<0.01, **p<0.001. EU: ELISA units. ED50: effective dose 50.
