# Supplementary figures and images for: Measurement of Human Papillomavirus-Specific Antibodies Using a Pseudovirion-Based ELISA Method
Source: Front Immunol. 2020 Oct 28;11:585768. doi: 10.3389/fimmu.2020.585768 (PMC7655971; doi:10.3389/fimmu.2020.585768)

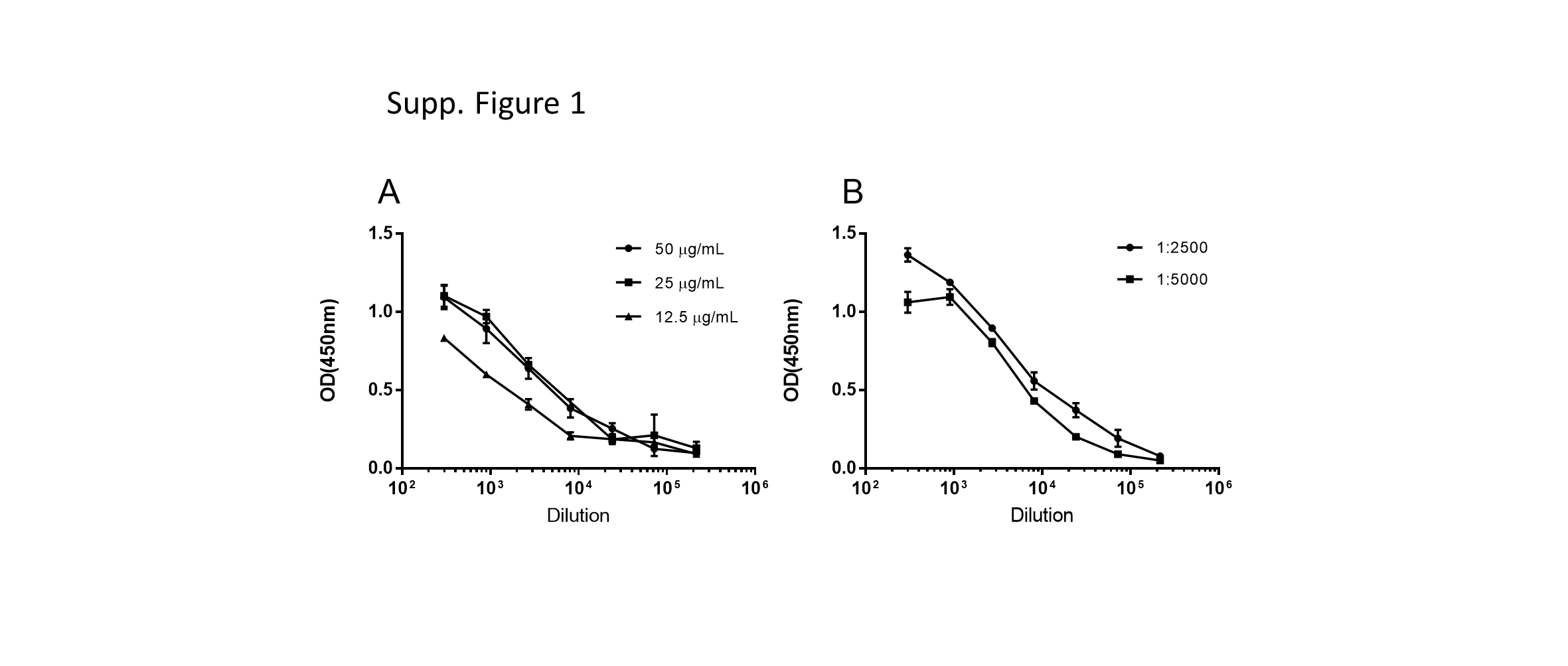

Supplement: Supplementary Figure 1 — Optimization conditions for antigen coating (A) and secondary antibody concentration (B) for PsV-ELISA using PsV-16. Pooled serum sample from HPV-vaccinated individuals had a starting dilution at 1:300. [file Image_1.tif]

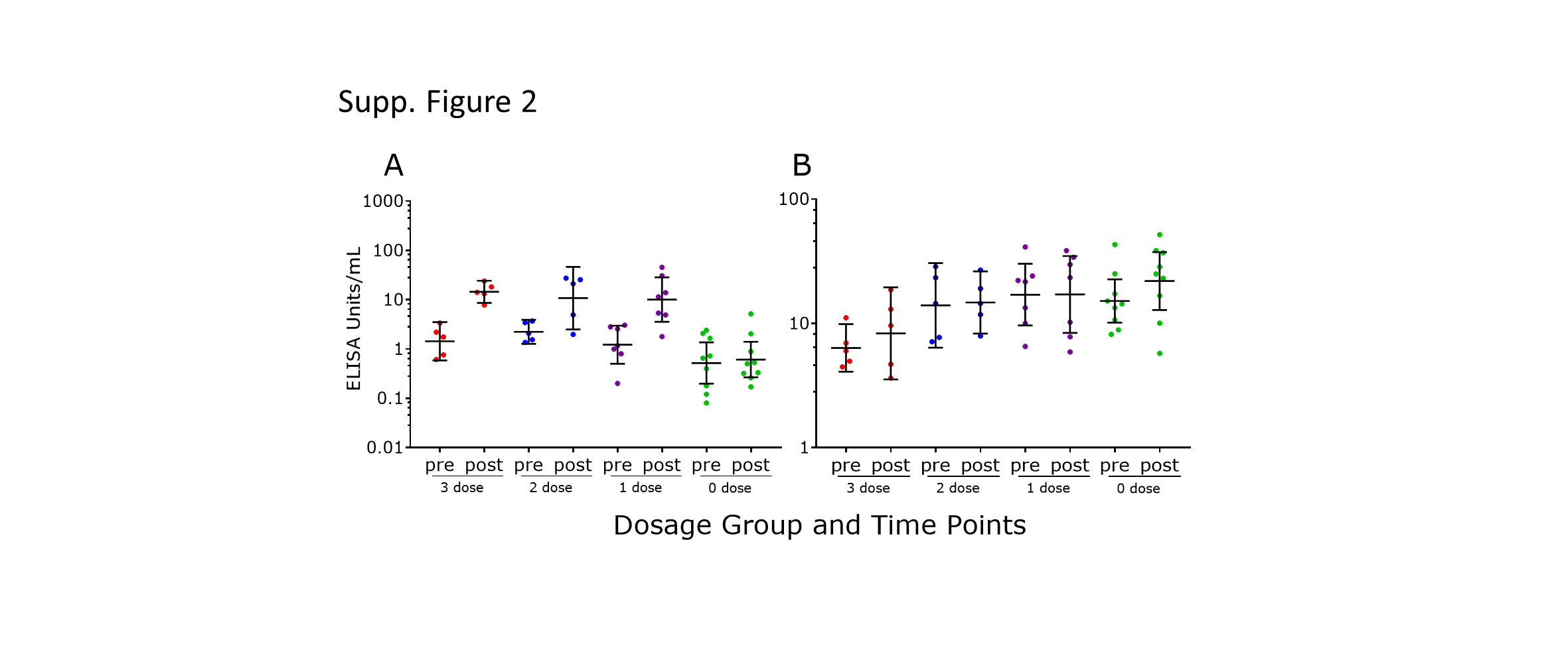

Supplement: Supplementary Figure 2 — HPV18-specific antibody responses measured by PsV-ELISA pre- and post-2vHPV. (A) HPV-specific IgG levels (B) HPV-specific IgM levels. Each dot represents an individual sample (3-dose group, N=5, 2-dose group, N=5, 1-dose group, N=7, and 0-dose group, N=9); error bars represent geometric mean ± 95% confidence interval. **p<0.01, **p<0.001. EU: ELISA units. ED50: effective dose 50. [file Image_2.tif]
